# Protease inhibitor plasma concentrations associate with COVID-19 infection

**DOI:** 10.1093/oxfimm/iqab014

**Published:** 2021-07-07

**Authors:** Nicholas R Medjeral-Thomas, Anne Troldborg, Annette G Hansen, Rasmus Pihl, Candice L Clarke, James E Peters, David C Thomas, Michelle Willicombe, Yaseelan Palarasah, Marina Botto, Matthew C Pickering, Steffen Thiel

**Affiliations:** 1 Centre for Inflammatory Disease, Imperial College London, UK; 2 Renal and Transplant Centre, Imperial College Healthcare NHS Trust, London, UK; 3 Department of Biomedicine, Aarhus University, Aarhus, Denmark; 4 Department of Rheumatology, Aarhus University Hospital, Aarhus, Denmark; 5 Chemical Biology Program, Memorial Sloan Kettering Cancer Center, New York, USA; 6 Department of Cancer & Inflammation Research, University of Southern Denmark, Odense, Denmark

**Keywords:** protease inhibitors, innate immunity, COVID-19, coronavirus

## Abstract

Protease inhibitors influence a range of innate immunity and inflammatory pathways. We quantified plasma concentrations of key anti-inflammatory protease inhibitors in chronic haemodialysis patients with coronavirus disease 2019 (COVID-19). The samples were collected early in the disease course to determine whether plasma protease inhibitor levels associated with the presence and severity of COVID-19. We used antibody-based immunoassays to measure plasma concentrations of C1 esterase inhibitor, alpha2-macroglobulin, antithrombin and inter-alpha-inhibitor heavy chain 4 (ITIH4) in 100 serial samples from 27 haemodialysis patients with COVID-19. ITIH4 was tested in two assays, one measuring intact ITIH4 and another also detecting any fragmented ITIH4 (total ITIH4). Control cohorts were 32 haemodialysis patients without COVID-19 and 32 healthy controls. We compared protease inhibitor concentration based on current and future COVID-19 severity and with C-reactive protein. Results were adjusted for repeated measures and multiple comparisons. Analysis of all available samples demonstrated lower plasma C1 esterase inhibitor and α2M and higher total ITIH4 in COVID-19 compared with dialysis controls. These differences were also seen in the first sample collected after COVID-19 diagnosis, a median of 4 days from diagnostic swab. Plasma ITIH4 levels were higher in severe than the non-severe COVID-19. Serum C-reactive protein correlated positively with plasma levels of antithrombin, intact ITIH4 and total ITIH4. In conclusion, plasma protease inhibitor concentrations are altered in COVID-19.

## INTRODUCTION

Infection by human severe acute respiratory syndrome coronavirus 2 (SARS-CoV-2) can lead to coronavirus disease 2019 (COVID-19) with clinical sequelae that range from mild symptoms to fatal pneumonitis. The immunological determinants of COVID-19 severity are not understood. Innate immune responses can influence COVID-19 susceptibility and severity by contributing to viral clearance and inflammation triggered by SARS-CoV-2 [1]. Severe COVID-19 is characterized by inflammatory and immuno-thrombotic pathway activation, many of which are regulated by protease inhibitors.

Inhibitors of plasma proteases often target multiple, distinct proteolytic events within immuno-thrombotic cascades, making them prime candidates for influencing the disease course of COVID-19. C1 esterase inhibitor (C1-INH, SERPING1) is a protease inhibitor that influences multiple innate immunity and inflammatory pathways [2]. In addition to inhibiting the complement classical and lectin pathways, C1-INH is a primary inhibitor of, e.g. activated coagulation pathway factors XII and XI, activated plasma kallikrein, plasmin, tissue-type plasminogen activator and thrombin [3]. C1-INH circulates at serum concentrations of 210–290 µg/ml in adults. Antithrombin III (AT, SERPINC1), alpha2-macroglobulin (α2M, A2M) and alpha1-antitrypsin (SERPINA1) are protease inhibitors that circulate at similarly abundant serum concentrations of ∼150, 140–410 and 1000–1500 µg/ml, respectively [4–6]. The newly characterized inter-alpha-inhibitor heavy chain 4 (ITIH4) has serum concentration of ∼226 µg/ml (Laursen *et al.*, manuscript in review). When ITIH4 is cleaved by an enzyme, such as lectin complement proteases and kallikrein, the cleaved ITIH4 fragment forms a non-covalent, inhibitory complex that inhibits the enzyme [7].

Mass spectrometry-based proteomic studies have identified associations between COVID-19 and protease inhibitors, including C1-INH, ITIH4, AT and alpha-1-antitrypsin [8–10]. SARS-CoV-2 proteins are predicted to interact with C1-INH and reduce C1-INH availability, which could contribute to inflammatory and pro-coagulant states observed in severe COVID-19 [11]. Reduced serum AT levels have been documented in patients with bacterial septicaemia, associated with markers of disseminated intravascular coagulation [12, 13], and have been observed in cohorts of critically ill patients with COVID-19 [14–16]. However, accurate circulating protease inhibitor concentrations quantified with targeted antibody-based assays have not been reported in COVID-19. Finally, the potential contribution of protease inhibitors to the pathogenesis of mild and recent-onset COVID-19 is not known.

Here we describe plasma levels of key protease inhibitors in patients with COVID-19, including individuals with mild disease. We collected serial samples during the first wave of the COVID-19 pandemic in London, the UK, from patients established on maintenance haemodialysis renal replacement therapy. Because of pre-existing chronic kidney disease, high comorbidity burden, relatively old age, and a high proportion of non-white ethnicity, the patient population was at increased risk of severe COVID-19 [14, 17–19]. Furthermore, the necessity to attend outpatient haemodialysis meant individuals were screened for pyrexia and symptoms of COVID-19 and subsequently diagnosed, enrolled and sampled at an early point in the disease course. We quantified plasma concentrations of C1-INH, AT, α2M and ITIH4, and identified multiple associations between protease inhibitor levels and COVID-19 severity.

## MATERIALS AND METHODS

All participants provided written informed consent and were enrolled in The Impact of COVID-19 on Renal and Immunosuppressed Patients study (IRAS ID 282077). The study was approved by the Health Research Authority, Research Ethics Committee (reference: 20/WA/0123) and conducted in accordance with the Declaration of Helsinki principles. We screened all individuals for symptoms and pyrexia at haemodialysis, clinic or emergency hospital attendance and tested individuals with SARS-CoV-2 nasopharyngeal PCR swab. We diagnosed COVID-19 from the date of the first positive SARS-CoV-2 PCR swab. We screened controls for asymptomatic COVID-19 infection with negative PCR swab and IgG assay for SARS-CoV-2 antibodies, as previously described [20]. Blood sampling commenced as soon as feasible after COVID-19 diagnosis.

Clinical data were collected from electronic medical records, anonymized and stored on secure computer networks at Imperial College Healthcare Trust. We defined COVID-19 severity based on World Health Organization (WHO) classifications (WHO clinical management of COVID-19: Interim guidance 27 May 2020. https://apps.who.int/iris/handle/10665/332196) adapted for clinical data availability. Mild was defined as COVID-19 symptoms but no evidence of pneumonia and no hypoxia. Moderate was defined as symptoms of pneumonia, but peripheral oxygen saturation (SaO2) >92% on air or an oxygen requirement no >4 l/min. Severe was defined as SaO2 <92% on air, respiratory rate >30/min or oxygen requirement >4 l/min. Critical was defined as organ dysfunction, signs of systemic shock, or the need for high dependency or intensive care support, e.g. non-invasive ventilation or intubation. Severity scores were charted throughout a patient's illness, including at each sampling point. For some analyses, we combined mild and moderate COVID-19 as ‘non-severe’ and severe and critical as ‘severe’.

We measured plasma C1-INH, AT, α2M and ITIH4 concentrations of 100 plasma samples from 27 patients with chronic kidney disease and COVID-19, and 1 sample each from 32 haemodialysis control patients without COVID-19 (dialysis controls) and 32 healthy volunteers with neither kidney disease nor COVID-19 (healthy controls), providing a total of 164 samples. Samples were taken at the start of haemodialysis treatment. Blood was collected in EDTA tubes and centrifuged to obtain plasma and stored at –80°C. Protease inhibitor concentrations were analysed in EDTA plasma with ‘in-house’ sandwich-type, antibody-based immunoassays designed and performed at Aarhus University, Denmark [7] or with the use of commercial antibody pairs (Supplemental methods). ITIH4 was tested in two assays; one detects intact ITIH4 only, the other also detects ITIH4 cleaved by enzymes (total ITIH4; Supplemental methods). We did not have access to an assay to measure alpha1-antitrypsin. Of the 100 COVID-19 samples, 63 were collected coincidentally with clinical samples for C-reactive protein (CRP).

Statistical analyses were performed using Graphpad Prism 9.0. Protein concentrations were displayed as mean with interquartile range (IQR). Differences in clinical characteristics were calculated with the Mann–Whitney *U* test for continuous and Fisher Exact tests for categorical data. Because our data included serial samples and different sample numbers in each cohort, we analysed these data with a mixed model that uses a compound symmetry covariance matrix and is fitted using Restricted Maximum Likelihood (REML). We adjusted the data for non-sphericity with the Geisser–Greenhouse correction. Differences between first sample lectin pathway concentrations were calculated with Kruskal–Wallis tests, follow-up comparison of the mean rank of every column, and adjustment of *P* values for multiple comparisons. We calculated correlations with Spearman’s rank test. We adjusted *P* -values for multiple comparisons using Bonferroni’s multiple comparisons test.

## RESULTS

In total, 11 of the 27 COVID-19 patients (41%) had severe disease (Table 1). Four patients (15%) died from COVID-19. Levels of clinical biomarkers associated with COVID-19, including CRP and D-dimer, were higher in the severe compared with non-severe disease cohorts (Table 1). Of the COVID-19 patients, 37% (10 of 27) were Asian and 22% (6 of 27) were of Black ethnicity (Table 1). Our COVID-19 patient population had a median age of 73 years (range 40–88 years), which was significantly older than the dialysis control (62 years, *P* = 0.004) and the healthy control (48 years, *P* < 0.0001) cohorts (Table 1).

**Table 1:** Characteristics of haemodialysis patients with COVID-19 and control cohorts

Category	Characteristic	COVID-19	Dialysis controls	Healthy controls	Severe COVID-19	Non-severe COVID-19	Difference	95% CI	*P*
	Number	27	32	32	11	16			
	Age, years	73 (range 40–88)	62 (range 19–86)^*^				11	4–19	0.004
				48 (range 28–63)^*^			24	18–30	<0.0001
					66 (44–88)	74 (40–84)			
	Male	17 (63)	19 (59)	17 (53)	7 (64)	10 (63)			
Ethnicity	BAME	18 (67)	24 (75)	20 (63)	6 (54)	12 (75)			
	Black	6 (22)	3 (9)	6 (19)	3 (27)	3 (18)			
	Asian	10 (37)	14 (44)	14 (44)	3 (27)	7 (47)			
	White	9 (33)	8 (25)	12 (37)	5 (46)	4 (24)			
	Other	2 (7)	7 (22)	0 (0)	0 (6)	2 (12)			
Kidney disease	Diabetic nephropathy	11 (41)	13 (41)		7 (44)	6 (35)			
	Hypertension	2 (7)	0 (0)		1 (6)	2 (12)			
	Glomerulonephritis	4 (14)	8 (25)		1 (6)	3 (18)			
	Genetic	1 (4)	1 (3)		1 (6)	1 (6)			
	Unknown	3 (11)	9 (28)		3 (19)	2 (12)			
	Other	6 (22)	1 (3)		3 (19)	3 (18)			
Co-morbidities	Ischaemic heart disease	14 (52)	15 (47)		7 (44)	10 (59)			
	Current smoking	0 (0)	2 (6)		0 (0)	0(0)			
	Ex-smoker	19 (70)	24 (75)		11 (69)	11 (65)			
	Type 2 diabetes mellitus	12 (44)	15 (47)		8 (50)	7 (41)			
	Antihypertensive medications	22 (81)	23 (72)		13 (81)	15 (88)			
	Current immunosuppression	5 (19)	2 (6)		4 (25)	4 (24)			
COVID-19 progression	Required hospitalization	11 (41)			16 (100)	1 (6)^**^			<0.0001
	Died from COVID-19	3 (11)			4 (25)	0 (0)^**^			0.04
Clinical biomarker at diagnostic swab	CRP. NR < 5 mg/l	43 (IQR 16–93)			60 (IQR 24–138)	29 (IQR 6–77)	31	−79 to 11	0.1
	D-dimer. NR <500 ng/ml	1818 (IQR 1087–2475)			1887 (IQR 1700–2973)	1479 (IQR 958–2064)	408	−1567 to 323	0.2
	Serum troponin. NR <34 ng/l	58 (IQR 27–104)			146 (IQR 63–168)	35 (IQR 22–65)^**^	111	15– 134	0.01
	Serum ferritin. NR 20-300 μg/l	841 (IQR 445–1531)			1938 (IQR 1241–2294)	520 (IQR 330–878) ^**^	1418	529–1772	0.0009
	White cell count. NR 4–11 × 10^9^/l	5.5 (IQR 3.8–6.2)			4.3 (IQR 2.9–6.0)	5.8 (IQR 4.3–6.6)	1.5	−0.6 to 2.7	0.2
	Lymphocyte count. NR 1–4 × 10^9^/l	0.9 (IQR 0.5–1.1)			0.5 (IQR 0.4–0.9)	1 (IQR 0.7–1.3) ^**^	−0.5	−0.7 to −0.1	0.03
Peak level of clinical biomarker	CRP. NR < 5 mg/l	124 (IQR 37–168)			171 (IQR 140–228)	40 (IQR 24–95) ^**^	131	91–192	<0.0001
	D-dimer. NR <500 ng/ml	1986 (IQR 1450–3552)			3464 (IQR 1864–4334)	1927 (IQR 1317–3005)^**^	1537	30–2844	0.049
	Serum troponin. NR <34 ng/l	69 (IQR 30–114)			152 (IQR 105–232)	40 (IQR 22–69)^**^	112	44–171	0.0004
	Serum ferritin. NR 20–300 μg/l	992 (IQR 639–2206)			2835 (IQR 1637–3408)	666 (IQR 543–938)^**^	2169	684–2646	0.0006
	White cell count. NR 4–11 × 10^9^/l	7.4 (IQR 5.9–9.4)			9.8 (IQR 7.7–11.1)	6.7 (IQR 5.6–7.5)^**^	3.1	0.9–5.1	0.006
	Lymphocyte count, nadir	0.7 (IQR 0.4–1.0)			0.4 (IQR 0.3–0.6)	0.9 (IQR 0.7–1.1)^**^	−0.5	−0.7 to −0.2	0.002

*Notes:* Data are numbers (%), median (range) or median (IQR). *Statistically significant differences between COVID-19 and dialysis control or healthy control cohorts. **Statistically significant differences between patients with severe and non-severe peak COVID-19 clinical severity. Differences calculated with the Mann–Whitney *U* test for continuous and Fisher Exact tests for categorical data.

We identified significantly greater plasma C1-INH and α2M levels in haemodialysis patients without COVID-19 than healthy controls (Supplementary table). Mean plasma C1-INH concentrations were 325 µg/ml in dialysis control compared with 127 µg/ml in healthy control cohorts (*P* = 0.0005). Mean plasma α2M concentrations were 1500 µg/ml in dialysis control compared with 866 µg/ml in healthy control cohorts (*P* < 0.0001). We used the dialysis control cohort for ongoing comparisons.

**Figure 1:**
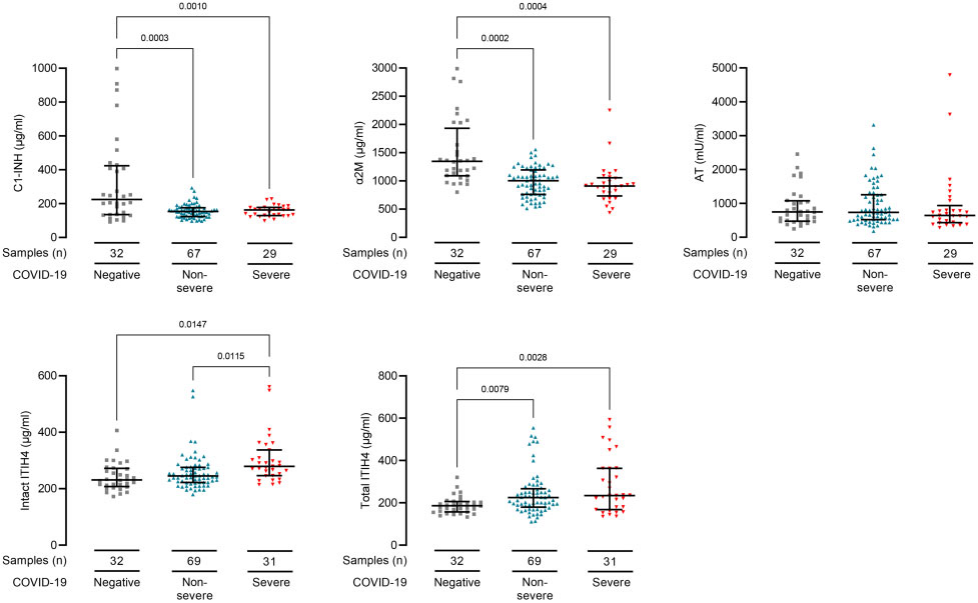
COVID-19 infection associates with reduced plasma C1-inhibitor, alfa2-macroglobulin, and increased ITIH4 levels. Plasma protease inhibitor levels in 100 samples from 27 haemodialysis patients with COVID-19. Overall, 31 samples were from patients with severe (red triangles) and 69 samples were from patients with non-severe (blue triangles) COVID-19 at sampling. Controls are 32 haemodialysis patients without COVID-19 (grey squares). Line and whiskers show the mean and standard deviation of the mean. Levels are shown in Supplemental Table S1. We analysed differences in protease inhibitor levels by fitting a mixed model in GraphPad Prism 8.0. This mixed model uses a compound symmetry covariance matrix and is fitted using REML. In the absence of missing values, this method gives the same *P* values and multiple comparisons tests as repeated measures ANOVA. In the presence of missing values, the results can be interpreted like repeated measures ANOVA. We adjusted the data for non-sphericity with the Geisser–Greenhouse correction. All *P*-values are adjusted with Bonferonni's multiple comparisons tests. ITIH4 was tested in two assays, one measuring intact ITIH4 only and another that in addition also detects any fragmented ITIH4 (total ITIH4).

We first determined whether protease inhibitor levels associated with COVID-19 diagnosis and severity at the time of sampling. This approach allowed us to utilize all samples and avoided sample selection bias. We identified lower plasma concentrations of C1-INH (*P* = 0.0003) and α2M (*P* = 0.0002) and higher plasma concentrations of total ITIH4 (*P* = 0.008) in COVID-19 samples (Fig. 1 and Supplementary table). Plasma intact ITIH4 levels were higher in samples from severe than non-severe COVID-19 (*P* = 0.02, Fig. 1 and Supplemental table). We did not identify differences between sub-cohorts of white, black and Asian ethnicity patients (data not shown).

**Figure 2:**
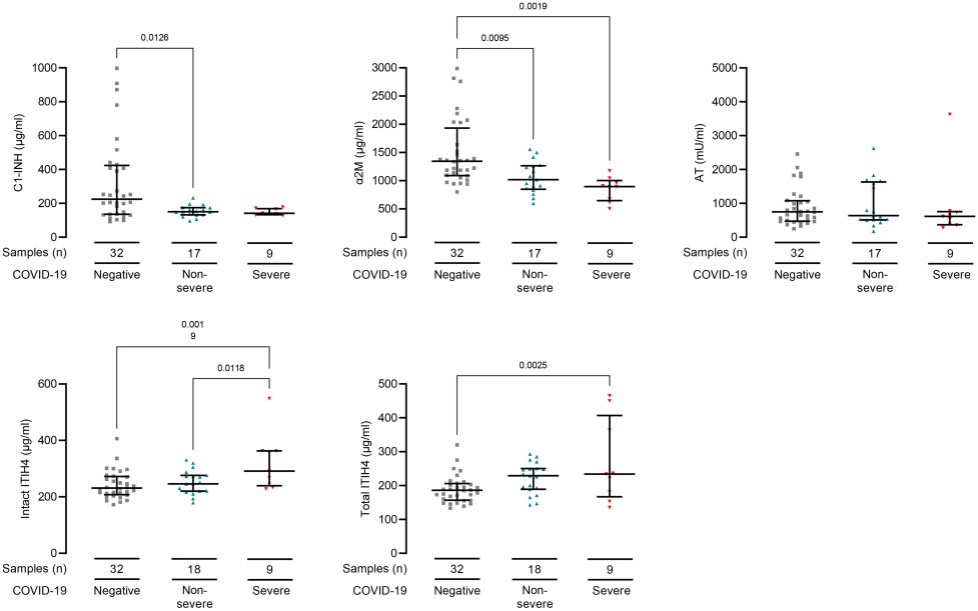
COVID-19 infection associates with reduced plasma C1-inhibitor, alfa2-macroglobulin and increased ITIH4 at first sampling point. Plasma protease inhibitor levels in first samples collected after COVID-19 diagnosis from 27 haemodialysis patients. Seventeen samples were from patients who developed severe disease (red triangles) and 9 samples were from patients with non-severe (blue triangles) COVID-19 only. Samples were collected at median 4 days (IQR: 2–10 days) from positive SARS-CoV2 swab and 6 days (IQR: 4–11 days) from symptom onset. Controls are 32 dialysis patients without COVID-19 (dialysis control cohort, grey squares). Line and whiskers show the mean and standard deviations. Differences in protease inhibitor levels were calculated by one-way ANOVA. All *P*-values are adjusted with Bonferonni's multiple comparisons tests. ITIH4 was tested in two assays, one measuring intact ITIH4 only and another that in addition also detects any fragmented ITIH4 (total ITIH4).

We identified similar associations between protease inhibitor concentrations and COVID-19 at the first sample collected after COVID-19 diagnosis. Plasma C1-INH (*P* = 0.01) and α2M (*P* = 0.001) were lower and total ITIH4 (*P* = 0.003) were higher in COVID-19 patients (Fig. 2). Intact ITIH4 was higher in patients who developed severe COVID-19 (*P* = 0.01, Fig. 2). These samples were collected at median 4 days (IQR: 2–10 days) from positive SARS-CoV2 swab and 6 days (IQR: 4–11 days) from symptom onset. These data demonstrate protease inhibitor concentrations are altered early in COVID-19 disease course.

**Figure 3:**
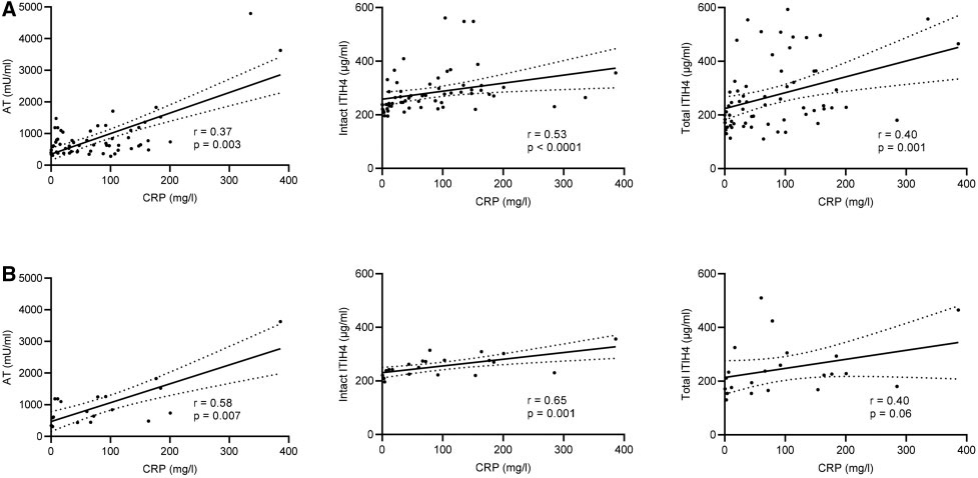
Associations between plasma protease inhibitor levels and CRP in COVID-19. Plasma protease inhibitor concentrations and serum CRP in samples from haemodialysis patients with COVID-19. (A) Shows all available samples (63 pairs) and (B) shows the first samples after COVID-19 diagnosis (22 pairs). Correlations (*r*) calculated with Spearman test. Solid lines show simple linear regression, and dotted lines show the 95% confidence intervals (CI). Significant correlations were not detected between CRP and either C1 esterase inhibitor or alfa2-macroglobulin (data not shown). ITIH4 was tested in two assays, one measuring intact ITIH4 only and another that in addition also detects any fragmented ITIH4 (total ITIH4).

We next examined if plasma protease inhibitor concentrations correlated with CRP because this clinical biomarker associates with active inflammation and severity of COVID-19 [22]. Plasma AT, intact ITIH4 and total ITHI4 correlated with serum CRP measured on coincidentally collected samples (Fig. 3A). Correlations between AT and intact ITHI4 and CRP were also significant when only the first collected samples post COVID-19 diagnosis were analysed (Fig. 3B).

## DISCUSSION

We demonstrated significant differences in protease inhibitor plasma concentrations in COVID-19. We identified reduced C1-INH and α2M and raised total ITIH4 levels in COVID-19 samples. Intact ITIH4 also associated with COVID-19 severity. In addition, AT and ITIH4 levels correlated positively with CRP, a biomarker of COVID-19 severity and inflammation [21]. Notably, the associations were also detectable in samples collected early in the disease course. These findings indicate that plasma protease inhibitors and inflammation interact both in early and established COVID-19. Accordingly, raised plasma ITIH4 could be developed as a biomarker of COVID-19 severity. Furthermore, the rebalancing of protease inhibitor levels, such as replenishing C1-INH or α2M, could be considered a therapeutic strategy for COVID-19. This is of particular relevance given the description of clinical improvement in four of five patients treated with human recombinant C1-INH for severe COVID-19 [22]. However, further research is needed to establish whether associations between protease inhibitor changes and COVID-19 are causative and to delineate the mechanisms that explain these associations.

Our data are consistent with data from mass spectrometry-based proteomic analyses of plasma and sera samples from COVID-19 patients. C1-INH was the protein with the most significantly reduced levels in samples from 31 COVID-19 patients compared with 262 controls [23]. From the same study, levels of ITIH4 were increased at first sampling in COVID-19 patients and inpatients who died from COVID-19 [24]. These results replicate associations between COVID-19 severity and C1-INH and ITIH4 identified from an isotope-labelled, targeted proteomic strategy applied to sera from 46 COVID-19 patients and 53 controls [10]. Increased ITIH4 levels were also demonstrated in a high throughput, mass spectrometry-based analysis of plasma and serum from 31 COVID-19 patients. This study also found increased C1r and C1s, the proteases inhibited by C1-INH, in COVID-19 [8]. Although these results from high protein coverage proteomic techniques are relevant, the accuracy of protein concentration measurements from proteomic approaches is limited [24]. In contrast, our assays are derived from targeted antibody-based techniques and provide robust quantification of protease inhibitor plasma concentrations. Furthermore, we tested ITIH4 with two assays, one that gives a signal from intact ITIH4 only and one that, in addition, provides a signal from cleaved ITIH4 (total ITIH4). Both assays detected differences between the dialysis controls and COVID-19 cohorts, whereas only the intact ITIH4 assay identified differences between non-severe and severe COVID-19.

We observed markedly increased plasma C1-INH and α2M levels in uninfected haemodialysis patients compared with healthy controls. Associations have been identified between elevated serum α2M and dialysis related amyloidosis [25, 26]. To our knowledge, circulating C1-INH has not been measured in haemodialysis patients previously. We do not know why plasma C1-INH is increased compared with healthy controls. Similar to α2M, these proteins could accumulate as a result of incomplete removal across semi-permeable dialysis membranes [26]. However, whether this is due to molecule size, charge, solubility or other factors requires further investigation. In addition, assessment of the functional activity of C1-INH, which we did not measure in our cohort, is needed in haemodialysis patients.

Because these proteins influence multiple homeostatic mechanisms, altered concentrations could contribute to the complex, inflammation-associated morbidity associated with chronic haemodialysis [27]. Furthermore, although these findings require further investigation, they are immediately relevant to research of C1-INH and α2M in individuals with kidney impairment, including patients with COVID-19. Recently, associations were identified between increased plasma C1-INH and COVID-19 RNAemia in samples from 123 hospitalized COVID-19 patients, 78 of whom required intensive care unit support [28]. On the basis of our data, these findings could be confounded by an increased burden of kidney impairment and renal replacement therapy in patients with high RNAemia and severe COVID-19.

In conclusion, we identified multiple associations between plasma levels of highly abundant protease inhibitors and COVID-19. Protease inhibitor plasma concentrations reflect and may influence COVID-19 pathology. Further research into the mechanistic interplay between protease inhibitor levels and COVID-19 pathogenesis is warranted to establish whether protease inhibitors are useful biomarkers and therapeutic targets for COVID-19.

## SUPPLEMENTARY DATA

Supplementary data is available at *Oxford Immunology* online.

## Supplementary Material

iqab014_Supplementary_Data

## References

[1] Schultze JL , Aschenbrenner AC. COVID-19 and the human innate immune system. Cell 2021;184:1671–92. 33743212 10.1016/j.cell.2021.02.029 PMC7885626

[2] Maas C , Oschatz C, Renne T. The plasma contact system 2.0. Semin Thromb Hemost 2011;37:375–81. 21805443 10.1055/s-0031-1276586

[3] Davis AE 3rd , Lu F, Mejia P. C1 inhibitor, a multi-functional serine protease inhibitor. Thromb Haemost 2010;104:886–93. 20806108 10.1160/TH10-01-0073

[4] Conard J , Brosstad F, Lie Larsen M et al Molar antithrombin concentration in normal human plasma. Haemostasis 1983;13:363–8. 6667903 10.1159/000214823

[5] Yoshino S , Fujimoto K, Takada T et al Molecular form and concentration of serum alpha2-macroglobulin in diabetes. Sci Rep 2019;9:12927. 31506491 10.1038/s41598-019-49144-7 PMC6736885

[6] Senn O , Russi EW, Schindler C et al Circulating alpha1-antitrypsin in the general population: determinants and association with lung function. Respir Res 2008;9:35. 18439253 10.1186/1465-9921-9-35 PMC2413219

[7] Pihl R , Jensen RK, Poulsen EC et al ITIH4 acts as a protease inhibitor by a novel inhibitory mechanism. Sci Adv 2021;7. 10.1126/sciadv.aba7381 PMC7793589 33523981

[8] Messner CB , Demichev V, Wendisch D et al Ultra-high-throughput clinical proteomics reveals classifiers of COVID-19 infection. Cell Syst 2020;11:11–24e4. 32619549 10.1016/j.cels.2020.05.012 PMC7264033

[9] D'Alessandro A , Thomas T, Dzieciatkowska M et al Serum proteomics in COVID-19 patients: altered coagulation and complement status as a function of IL-6 level. J Proteome Res 2020;19:4417–27. 32786691 10.1021/acs.jproteome.0c00365 PMC7640953

[10] Shen B , Yi X, Sun Y et al Proteomic and metabolomic characterization of COVID-19 patient sera. Cell 2020;182:59–72 e15. 32492406 10.1016/j.cell.2020.05.032 PMC7254001

[11] Thomson TM , Toscano-Guerra E, Casis E et al C1 esterase inhibitor and the contact system in COVID-19. Br J Haematol 2020;190:520–4. 32531085 10.1111/bjh.16938 PMC7323335

[12] Fourrier F , Chopin C, Goudemand J et al Septic shock, multiple organ failure, and disseminated intravascular coagulation. Compared patterns of antithrombin III, protein C, and protein S deficiencies. Chest 1992;101:816–23. 1531791 10.1378/chest.101.3.816

[13] Mesters RM , Mannucci PM, Coppola R et al Factor VIIa and antithrombin III activity during severe sepsis and septic shock in neutropenic patients. Blood 1996;88:881–6. 8704245

[14] Zhou F , Yu T, Du R et al Clinical course and risk factors for mortality of adult inpatients with COVID-19 in Wuhan, China: a retrospective cohort study. Lancet 2020;395:1054–62. 32171076 10.1016/S0140-6736(20)30566-3 PMC7270627

[15] Cui S , Chen S, Li X et al Prevalence of venous thromboembolism in patients with severe novel coronavirus pneumonia. J Thromb Haemost 2020;18:1421–4. 32271988 10.1111/jth.14830 PMC7262324

[16] Huang C , Wang Y, Li X et al Clinical features of patients infected with 2019 novel coronavirus in Wuhan, China. Lancet 2020;395:497–506. 31986264 10.1016/S0140-6736(20)30183-5 PMC7159299

[17] Wu Z , McGoogan JM. Characteristics of and important lessons from the coronavirus Disease 2019 (COVID-19) outbreak in China: summary of a report of 72314 cases from the Chinese Center for disease control and prevention. J Am Med Assoc 2020;323:1239–42. 10.1001/jama.2020.2648 32091533

[18] Williamson EJ , Walker AJ, Bhaskaran K et al Factors associated with COVID-19-related death using OpenSAFELY. Nature 2020;584:430–6. 32640463 10.1038/s41586-020-2521-4 PMC7611074

[19] Cheng Y , Luo R, Wang K et al Kidney disease is associated with in-hospital death of patients with COVID-19. Kidney Int 2020;97:829–38. 32247631 10.1016/j.kint.2020.03.005 PMC7110296

[20] Clarke C , Prendecki M, Dhutia A et al High prevalence of asymptomatic COVID-19 infection in haemodialysis patients detected using serologic screening. J Am Soc Nephrol 2020;31:1969–75. 32732391 10.1681/ASN.2020060827 PMC7461667

[21] Henry BM , de Oliveira MHS, Benoit S et al Haematologic, biochemical and immune biomarker abnormalities associated with severe illness and mortality in coronavirus disease 2019 (COVID-19): a meta-analysis. Clin Chem Lab Med 2020;58:1021–8. 32286245 10.1515/cclm-2020-0369

[22] Urwyler P , Moser S, Charitos P et al Treatment of COVID-19 with conestat alfa, a regulator of the complement, contact activation and kallikrein-kinin system. Front Immunol 2020;11:2072. 32922409 10.3389/fimmu.2020.02072 PMC7456998

[23] Geyer PE , Arend FM, Doll S et al High-resolution longitudinal serum proteome trajectories in COVID-19 reveal patients-specific seroconversion. medRxiv 2021; doi:10.1101/2021.02.22.21252236. PMC8687121 34232570

[24] Kinoshita Y , Uo T, Jayadev S et al Potential applications and limitations of proteomics in the study of neurological disease. Arch Neurol 2006;63:1692–6. 17172608 10.1001/archneur.63.12.1692

[25] Argiles A , Kerr PG, Mourad G et al Serum alpha 2-macroglobulin in haemodialysis patients: baseline and kinetic studies. Nephrol Dial Transplant. 1993;8:1118–23. 7505905

[26] Motomiya Y , Ando Y, Haraoka K et al Circulating level of alpha2-macroglobulin-beta2-microglobulin complex in haemodialysis patients. Kidney Int 2003;64:2244–52. 14633149 10.1046/j.1523-1755.2003.00315.x

[27] Jofre R , Rodriguez-Benitez P, Lopez-Gomez JM et al Inflammatory syndrome in patients on haemodialysis. J Am Soc Nephrol 2006;17:S274–80. 17130274 10.1681/ASN.2006080926

[28] Gutmann C , Takov K, Burnap SA et al SARS-CoV-2 RNAemia and proteomic trajectories inform prognostication in COVID-19 patients admitted to intensive care. Nat Commun 2021;12:3406. 34099652 10.1038/s41467-021-23494-1 PMC8184784

