# Supplementary material for: Protease inhibitor plasma concentrations associate with COVID-19 infection
Source: Oxf Open Immunol. 2021 Jul 7;2(1):iqab014. doi: 10.1093/oxfimm/iqab014 (PMC8371939; doi:10.1093/oxfimm/iqab014)
Supplement: iqab014_Supplementary_Data [file iqab014_supplementary_data.pptx]

## Slide 1
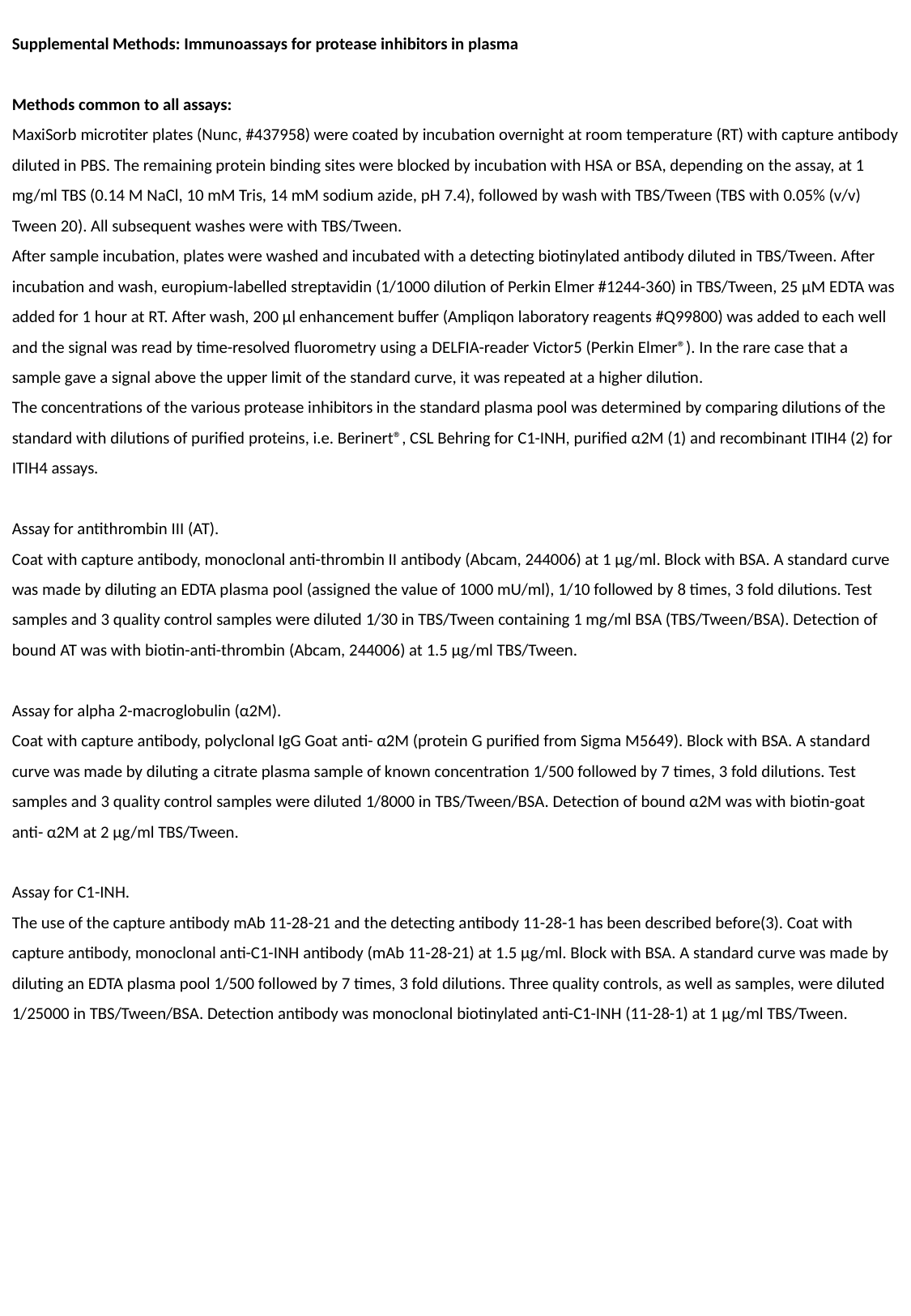

Supplemental Methods: Immunoassays for protease inhibitors in plasma
Methods common to all assays:
MaxiSorb microtiter plates (Nunc, #437958) were coated by incubation overnight at room temperature (RT) with capture antibody diluted in PBS. The remaining protein binding sites were blocked by incubation with HSA or BSA, depending on the assay, at 1 mg/ml TBS (0.14 M NaCl, 10 mM Tris, 14 mM sodium azide, pH 7.4), followed by wash with TBS/Tween (TBS with 0.05% (v/v) Tween 20). All subsequent washes were with TBS/Tween.
After sample incubation, plates were washed and incubated with a detecting biotinylated antibody diluted in TBS/Tween. After incubation and wash, europium-labelled streptavidin (1/1000 dilution of Perkin Elmer #1244-360) in TBS/Tween, 25 μM EDTA was added for 1 hour at RT. After wash, 200 μl enhancement buffer (Ampliqon laboratory reagents #Q99800) was added to each well and the signal was read by time-resolved fluorometry using a DELFIA-reader Victor5 (Perkin Elmer®). In the rare case that a sample gave a signal above the upper limit of the standard curve, it was repeated at a higher dilution.
The concentrations of the various protease inhibitors in the standard plasma pool was determined by comparing dilutions of the standard with dilutions of purified proteins, i.e. Berinert®, CSL Behring for C1-INH, purified α2M (1) and recombinant ITIH4 (2) for ITIH4 assays.
Assay for antithrombin III (AT).
Coat with capture antibody, monoclonal anti-thrombin II antibody (Abcam, 244006) at 1 µg/ml. Block with BSA. A standard curve was made by diluting an EDTA plasma pool (assigned the value of 1000 mU/ml), 1/10 followed by 8 times, 3 fold dilutions. Test samples and 3 quality control samples were diluted 1/30 in TBS/Tween containing 1 mg/ml BSA (TBS/Tween/BSA). Detection of bound AT was with biotin-anti-thrombin (Abcam, 244006) at 1.5 µg/ml TBS/Tween.
Assay for alpha 2-macroglobulin (α2M).
Coat with capture antibody, polyclonal IgG Goat anti- α2M (protein G purified from Sigma M5649). Block with BSA. A standard curve was made by diluting a citrate plasma sample of known concentration 1/500 followed by 7 times, 3 fold dilutions. Test samples and 3 quality control samples were diluted 1/8000 in TBS/Tween/BSA. Detection of bound α2M was with biotin-goat anti- α2M at 2 µg/ml TBS/Tween.
Assay for C1-INH.
The use of the capture antibody mAb 11-28-21 and the detecting antibody 11-28-1 has been described before(3). Coat with capture antibody, monoclonal anti-C1-INH antibody (mAb 11-28-21) at 1.5 µg/ml. Block with BSA. A standard curve was made by diluting an EDTA plasma pool 1/500 followed by 7 times, 3 fold dilutions. Three quality controls, as well as samples, were diluted 1/25000 in TBS/Tween/BSA. Detection antibody was monoclonal biotinylated anti-C1-INH (11-28-1) at 1 µg/ml TBS/Tween.

## Slide 2
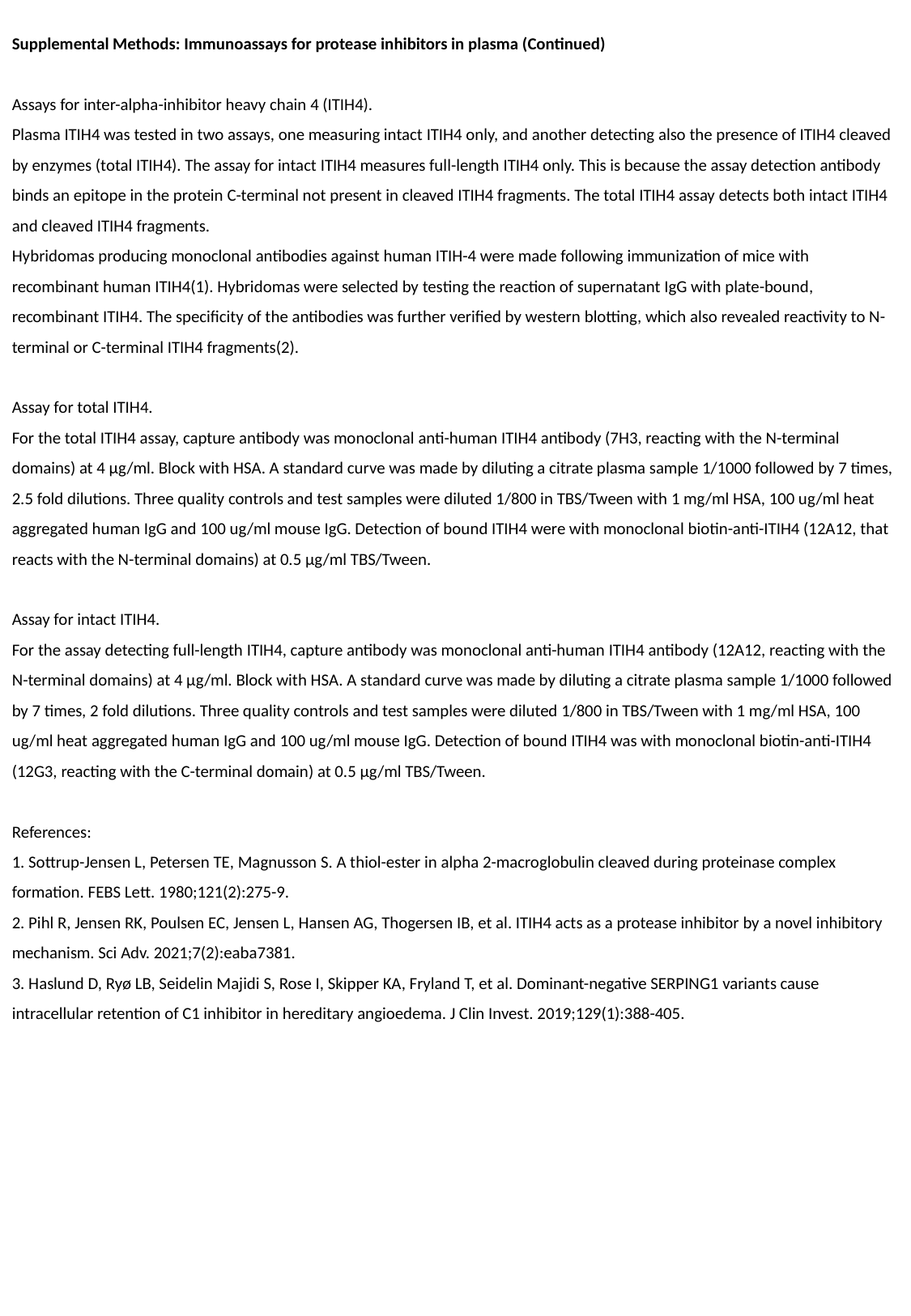

Supplemental Methods: Immunoassays for protease inhibitors in plasma (Continued)
Assays for inter-alpha-inhibitor heavy chain 4 (ITIH4).
Plasma ITIH4 was tested in two assays, one measuring intact ITIH4 only, and another detecting also the presence of ITIH4 cleaved by enzymes (total ITIH4). The assay for intact ITIH4 measures full-length ITIH4 only. This is because the assay detection antibody binds an epitope in the protein C-terminal not present in cleaved ITIH4 fragments. The total ITIH4 assay detects both intact ITIH4 and cleaved ITIH4 fragments.
Hybridomas producing monoclonal antibodies against human ITIH-4 were made following immunization of mice with recombinant human ITIH4(1). Hybridomas were selected by testing the reaction of supernatant IgG with plate-bound, recombinant ITIH4. The specificity of the antibodies was further verified by western blotting, which also revealed reactivity to N-terminal or C-terminal ITIH4 fragments(2).
Assay for total ITIH4.
For the total ITIH4 assay, capture antibody was monoclonal anti-human ITIH4 antibody (7H3, reacting with the N-terminal domains) at 4 µg/ml. Block with HSA. A standard curve was made by diluting a citrate plasma sample 1/1000 followed by 7 times, 2.5 fold dilutions. Three quality controls and test samples were diluted 1/800 in TBS/Tween with 1 mg/ml HSA, 100 ug/ml heat aggregated human IgG and 100 ug/ml mouse IgG. Detection of bound ITIH4 were with monoclonal biotin-anti-ITIH4 (12A12, that reacts with the N-terminal domains) at 0.5 µg/ml TBS/Tween.
Assay for intact ITIH4.
For the assay detecting full-length ITIH4, capture antibody was monoclonal anti-human ITIH4 antibody (12A12, reacting with the N-terminal domains) at 4 µg/ml. Block with HSA. A standard curve was made by diluting a citrate plasma sample 1/1000 followed by 7 times, 2 fold dilutions. Three quality controls and test samples were diluted 1/800 in TBS/Tween with 1 mg/ml HSA, 100 ug/ml heat aggregated human IgG and 100 ug/ml mouse IgG. Detection of bound ITIH4 was with monoclonal biotin-anti-ITIH4 (12G3, reacting with the C-terminal domain) at 0.5 µg/ml TBS/Tween.
References:
1. Sottrup-Jensen L, Petersen TE, Magnusson S. A thiol-ester in alpha 2-macroglobulin cleaved during proteinase complex formation. FEBS Lett. 1980;121(2):275-9.
2. Pihl R, Jensen RK, Poulsen EC, Jensen L, Hansen AG, Thogersen IB, et al. ITIH4 acts as a protease inhibitor by a novel inhibitory mechanism. Sci Adv. 2021;7(2):eaba7381.
3. Haslund D, Ryø LB, Seidelin Majidi S, Rose I, Skipper KA, Fryland T, et al. Dominant-negative SERPING1 variants cause intracellular retention of C1 inhibitor in hereditary angioedema. J Clin Invest. 2019;129(1):388-405.

## Slide 3
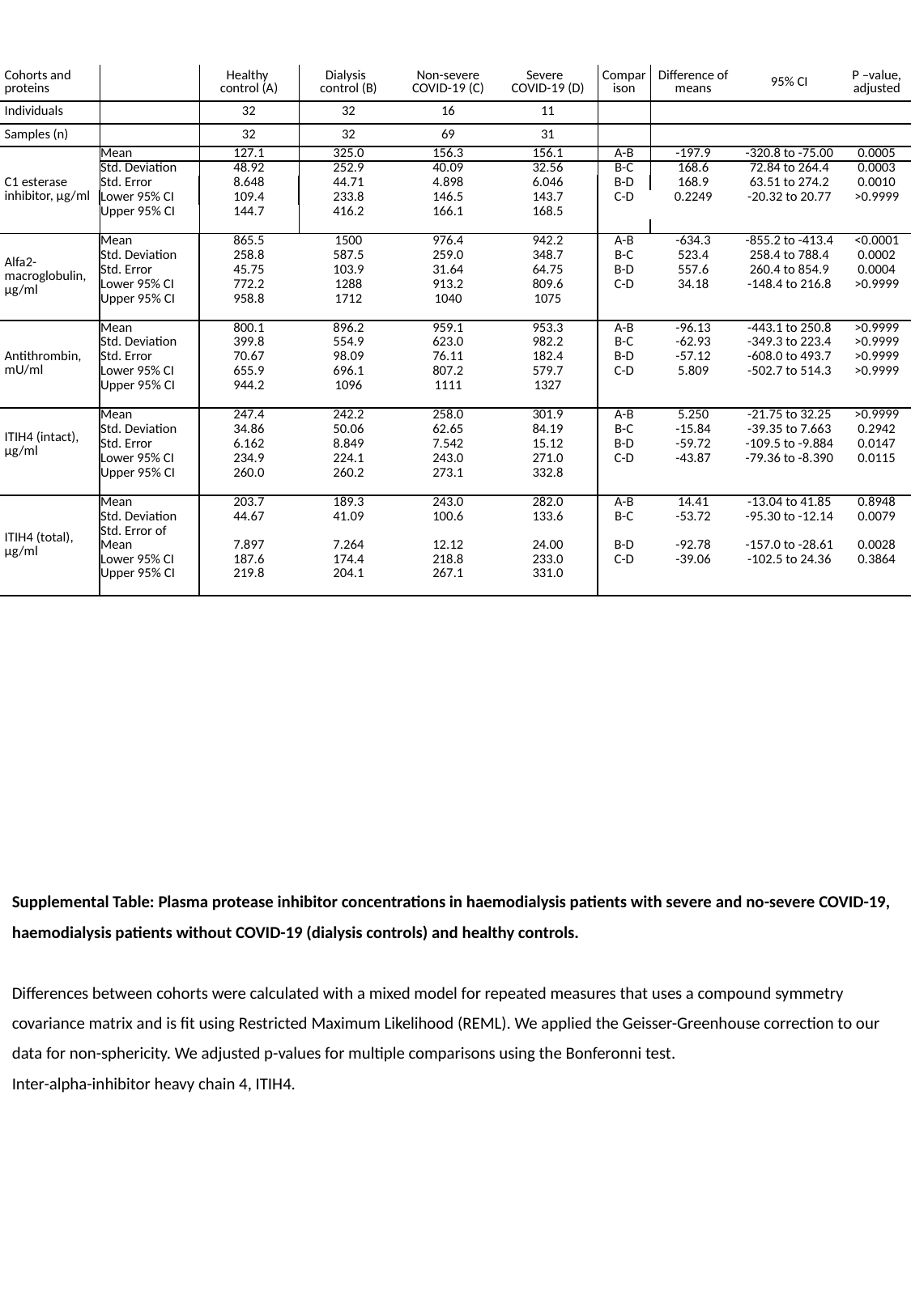

| Cohorts and proteins | | Healthy control (A) | Dialysis control (B) | Non-severe COVID-19 (C) | Severe COVID-19 (D) | Comparison | Difference of means | 95% CI | P –value, adjusted |
| --- | --- | --- | --- | --- | --- | --- | --- | --- | --- |
| Individuals | | 32 | 32 | 16 | 11 | | | | |
| Samples (n) | | 32 | 32 | 69 | 31 | | | | |
| C1 esterase inhibitor, µg/ml | Mean | 127.1 | 325.0 | 156.3 | 156.1 | A-B | -197.9 | -320.8 to -75.00 | 0.0005 |
| | Std. Deviation | 48.92 | 252.9 | 40.09 | 32.56 | B-C | 168.6 | 72.84 to 264.4 | 0.0003 |
| | Std. Error | 8.648 | 44.71 | 4.898 | 6.046 | B-D | 168.9 | 63.51 to 274.2 | 0.0010 |
| | Lower 95% CI | 109.4 | 233.8 | 146.5 | 143.7 | C-D | 0.2249 | -20.32 to 20.77 | >0.9999 |
| | Upper 95% CI | 144.7 | 416.2 | 166.1 | 168.5 | | | | |
| | | | | | | | | | |
| Alfa2-macroglobulin, µg/ml | Mean | 865.5 | 1500 | 976.4 | 942.2 | A-B | -634.3 | -855.2 to -413.4 | <0.0001 |
| | Std. Deviation | 258.8 | 587.5 | 259.0 | 348.7 | B-C | 523.4 | 258.4 to 788.4 | 0.0002 |
| | Std. Error | 45.75 | 103.9 | 31.64 | 64.75 | B-D | 557.6 | 260.4 to 854.9 | 0.0004 |
| | Lower 95% CI | 772.2 | 1288 | 913.2 | 809.6 | C-D | 34.18 | -148.4 to 216.8 | >0.9999 |
| | Upper 95% CI | 958.8 | 1712 | 1040 | 1075 | | | | |
| | | | | | | | | | |
| Antithrombin, mU/ml | Mean | 800.1 | 896.2 | 959.1 | 953.3 | A-B | -96.13 | -443.1 to 250.8 | >0.9999 |
| | Std. Deviation | 399.8 | 554.9 | 623.0 | 982.2 | B-C | -62.93 | -349.3 to 223.4 | >0.9999 |
| | Std. Error | 70.67 | 98.09 | 76.11 | 182.4 | B-D | -57.12 | -608.0 to 493.7 | >0.9999 |
| | Lower 95% CI | 655.9 | 696.1 | 807.2 | 579.7 | C-D | 5.809 | -502.7 to 514.3 | >0.9999 |
| | Upper 95% CI | 944.2 | 1096 | 1111 | 1327 | | | | |
| | | | | | | | | | |
| ITIH4 (intact), µg/ml | Mean | 247.4 | 242.2 | 258.0 | 301.9 | A-B | 5.250 | -21.75 to 32.25 | >0.9999 |
| | Std. Deviation | 34.86 | 50.06 | 62.65 | 84.19 | B-C | -15.84 | -39.35 to 7.663 | 0.2942 |
| | Std. Error | 6.162 | 8.849 | 7.542 | 15.12 | B-D | -59.72 | -109.5 to -9.884 | 0.0147 |
| | Lower 95% CI | 234.9 | 224.1 | 243.0 | 271.0 | C-D | -43.87 | -79.36 to -8.390 | 0.0115 |
| | Upper 95% CI | 260.0 | 260.2 | 273.1 | 332.8 | | | | |
| | | | | | | | | | |
| ITIH4 (total), µg/ml | Mean | 203.7 | 189.3 | 243.0 | 282.0 | A-B | 14.41 | -13.04 to 41.85 | 0.8948 |
| | Std. Deviation | 44.67 | 41.09 | 100.6 | 133.6 | B-C | -53.72 | -95.30 to -12.14 | 0.0079 |
| | Std. Error of Mean | 7.897 | 7.264 | 12.12 | 24.00 | B-D | -92.78 | -157.0 to -28.61 | 0.0028 |
| | Lower 95% CI | 187.6 | 174.4 | 218.8 | 233.0 | C-D | -39.06 | -102.5 to 24.36 | 0.3864 |
| | Upper 95% CI | 219.8 | 204.1 | 267.1 | 331.0 | | | | |
| | | | | | | | | | |
Supplemental Table: Plasma protease inhibitor concentrations in haemodialysis patients with severe and no-severe COVID-19, haemodialysis patients without COVID-19 (dialysis controls) and healthy controls.
Differences between cohorts were calculated with a mixed model for repeated measures that uses a compound symmetry covariance matrix and is fit using Restricted Maximum Likelihood (REML). We applied the Geisser-Greenhouse correction to our data for non-sphericity. We adjusted p-values for multiple comparisons using the Bonferonni test.
Inter-alpha-inhibitor heavy chain 4, ITIH4.

## Slide 4
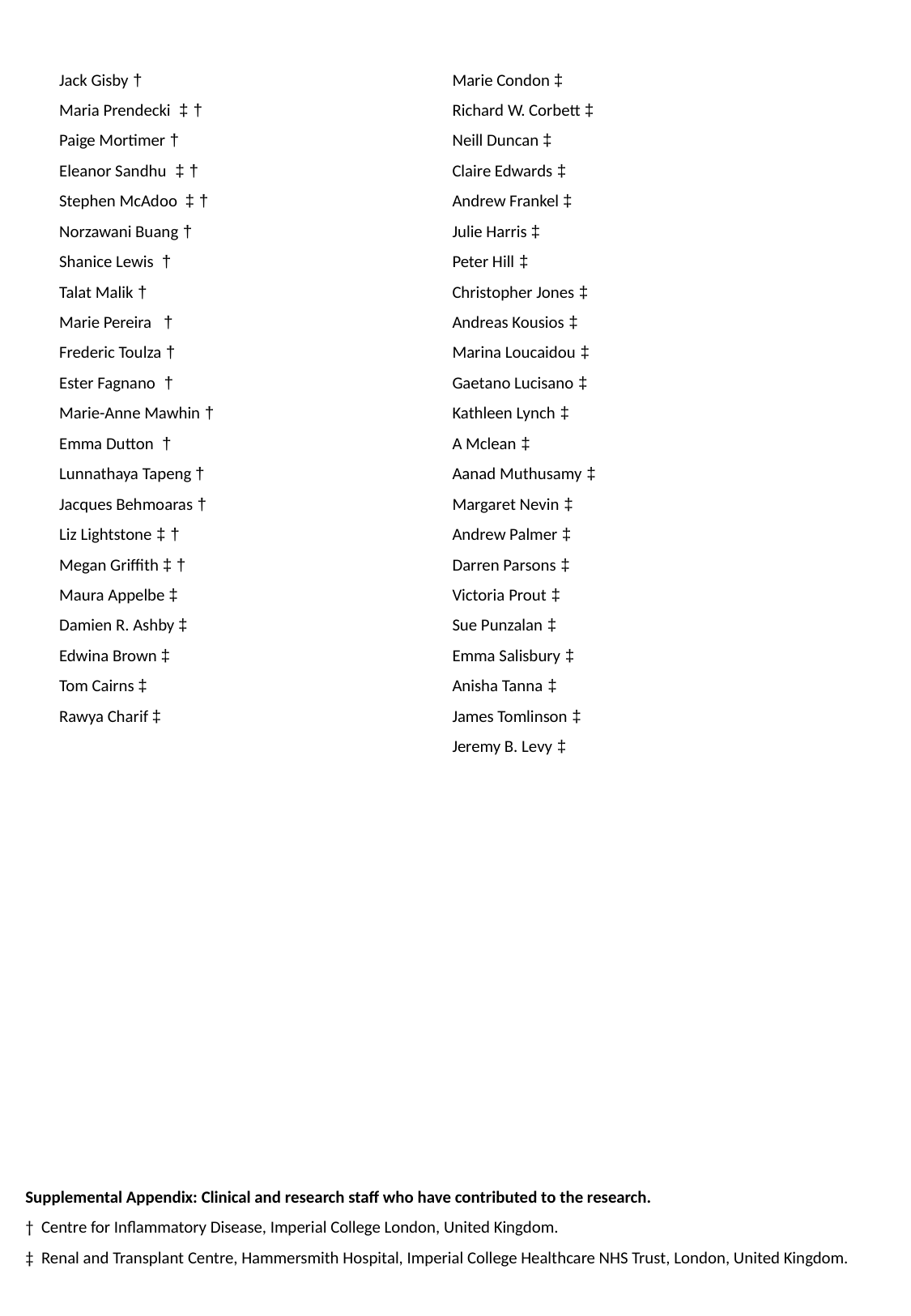

Jack Gisby †
Maria Prendecki  ‡ †
Paige Mortimer †
Eleanor Sandhu  ‡ †
Stephen McAdoo  ‡ †
Norzawani Buang †
Shanice Lewis  †
Talat Malik †
Marie Pereira   †
Frederic Toulza †
Ester Fagnano  †
Marie-Anne Mawhin †
Emma Dutton  †
Lunnathaya Tapeng †
Jacques Behmoaras †
Liz Lightstone ‡ †
Megan Griffith ‡ †
Maura Appelbe ‡
Damien R. Ashby ‡
Edwina Brown ‡
Tom Cairns ‡
Rawya Charif ‡
Marie Condon ‡
Richard W. Corbett ‡
Neill Duncan ‡
Claire Edwards ‡
Andrew Frankel ‡
Julie Harris ‡
Peter Hill ‡
Christopher Jones ‡
Andreas Kousios ‡
Marina Loucaidou ‡
Gaetano Lucisano ‡
Kathleen Lynch ‡
A Mclean ‡
Aanad Muthusamy ‡
Margaret Nevin ‡
Andrew Palmer ‡
Darren Parsons ‡
Victoria Prout ‡
Sue Punzalan ‡
Emma Salisbury ‡
Anisha Tanna ‡
James Tomlinson ‡
Jeremy B. Levy ‡
Supplemental Appendix: Clinical and research staff who have contributed to the research.
† Centre for Inflammatory Disease, Imperial College London, United Kingdom.
‡  Renal and Transplant Centre, Hammersmith Hospital, Imperial College Healthcare NHS Trust, London, United Kingdom.
